# A virtual experimenter does not increase placebo hypoalgesia when delivering an interactive expectancy manipulation

**DOI:** 10.1038/s41598-020-77453-9

**Published:** 2020-11-23

**Authors:** Bjoern Horing, Sarah C. Beadle, Zachariah Inks, Andrew Robb, Eric R. Muth, Sabarish V. Babu

**Affiliations:** 1 Affective Neuroscience Group, Department of Systems Neuroscience, University Medical Center Hamburg-Eppendorf, Martinistr. 52, 20246 Hamburg, Germany; 2 Department of Psychology, Clemson University, Clemson, SC USA; 3 Division of Human Centered Computing, School of Computing, Clemson University, Clemson, SC USA; 4 Division of Research and Economic Development, North Carolina Agricultural and Technical State University, Greensboro, NC USA

**Keywords:** Human behaviour, Medical research

## Abstract

Lack of standardization and unblinding threaten the research of mechanisms involved in expectancy effects on pain. We evaluated a computer-controlled virtual experimenter (VEx) to avoid these issues. Fifty-four subjects underwent a baseline-retest heat pain protocol. Between sessions, they received an expectancy manipulation (placebo or no-treatment) delivered by VEx or text-only control condition. The VEx provided standardized “social” interaction with the subjects. Pain ratings and psychological state/trait measures were recorded. We found an interaction of expectancy and delivery on pain improvement following the intervention. In the text conditions, placebo was followed by lower pain, whereas in the VEx conditions, placebo and no-treatment were followed by a comparable pain decrease. Secondary analyses indicated that this interaction was mirrored by decreases of negative mood and anxiety. Furthermore, changes in continuous pain were moderated by expectation of pain relief. However, retrospective pain ratings show an effect of expectancy but not of delivery. We conclude that we successfully applied an automated protocol for inducing expectancy effects on pain. The effect of the VEx regardless of treatment may be due to interactions of attention allocation and locus of control. This points to the diversity of expectancy mechanisms, and has implications for research and computer-based treatment applications.

## Introduction

Pain is a challenge to human well-being owing to its high prevalence as a clinical symptom^1^, corresponding societal costs and substantial impact on quality of life^2^. In many painful clinical conditions and experimental pain modalities, placebo effects account for a large amount of symptom change following interventions^3–5^. Because new treatments perform against placebo conditions, it is desirable that the variance attributable to placebo effects is as low as possible to better detect the efficacy of treatments^6^. Here, we report results using a virtual experimenter (VEx) to standardize interventions to more reliably control variance attributable to placebo effects.

Placebo effects are mediated by expectancies^6^—since symptom changes can be positive or negative (as in nocebo effects), these can more generally be termed “expectancy effects”. Unless a treatment is not perceived^7^, expectancy effects are implicated in *every* treatment, regardless of whether it involves an “active” component^5,6^. Expectancies arise from the complex interaction of information delivered alongside and through a treatment (for example, assertions of efficacy or potential side effects), explicit preconceptions about that treatment (for example, attitudes towards the treatment modality, such as acupuncture or medication), implicit preconceptions (for example, prior experiences, including conditioning)^8^, as well as mindset, mood and other determinants^5^.

When social interactions are involved, several related concepts, such as sympathy or trust, codetermine treatment outcome to a large degree^9^. For example, a pleasant interaction could per se elevate mood and well-being of a person (direct psychosocial effect), or it could facilitate adherence or the acceptance of treatment instructions (indirect psychosocial effect). Negative expectancies, such as those mediated by doubts about the intentions or the competence of a physician, could reduce or even abolish treatment effects^10^, or lead to an increased reporting of side effects^11^.

Biomedical research, and particularly research on expectancy effects, faces several methodological challenges. Two of these challenges are standardization and replicability^12,13^, which is difficult to achieve in psychosocial interactions, and blinding^14^, that is, that subjects and experimenters are unaware of whether the subject has been assigned to treatment or control group. Failure to address these challenges introduces systematic error that can render experimental results uninterpretable, such as self-fulfilling prophecies and implicit biases^15–17^. As we have argued before^18^, automating the tasks of experimental interactions and treatment allocation with a computerized experimenter could overcome some of these challenges.

Virtual reality simulations have been used as interventions before (e.g. as exposure therapy, or for digital pain management^19–23^), but not to address the breakpoints involved in human–human interaction especially in clinical studies. Our research indicates that standardization benefits from the introduction of virtual humans, for example in the identification of suspects in police line-ups presented to eye-witnesses, or in training novice healthcare providers in patient interviewing via standardized virtual patients^24,25^. Here, we followed our research agenda laid out before^18^ to explore a virtual experimenter’s (VEx) efficacy to avoid biases during the induction of positive expectancies in subjects undergoing a heat pain protocol, compared with a text/audio control condition. We employed a placebo hypoalgesia paradigm^18^, including an inert pill presented as an effective analgesic. We hypothesized that the expectancies should lead to lower pain reports, compared to the no-treatment control. Furthermore, we expected an interaction effect such that in the VEx condition, the impact of psychosocial factors would be more pronounced, facilitating expectancy effects on pain reporting.

## Methods

### Subjects

Fifty-four healthy volunteers (sex 29f:25m, age 19.6 ± 2.6) were recruited from Clemson University’s student population through word of mouth and an online recruitment system; sample size calculation is provided in Supplementary Text S1. All methods (recruitment and experiment) were carried out in accordance with relevant guidelines and regulations. Subjects filled out an online screening and survey. Inclusion criteria assessed online were: between 18 and 35 years of age, absence of past or present brain or nerve conditions or pain disorders, absence of open wounds or skin conditions on the dominant hand, and abstinence from pain medication and recreational drugs for at least 24 h before the lab visit. Any regular medication was also documented. Furthermore, subjects were only included below the clinical cutoffs provided by the Patient Health Questionnaire for depression (PHQ9 < 11) and somatization disorder (PHQ15 < 11)^26,27^.

Following screening, subjects were scheduled for the lab visit. The lab visit included another short survey, two pain measurement sessions (baseline and retest) and the experimental manipulation. Experiments were conducted from August 2017 through February 2018.

### Experimental design and overview

**Figure 1:**
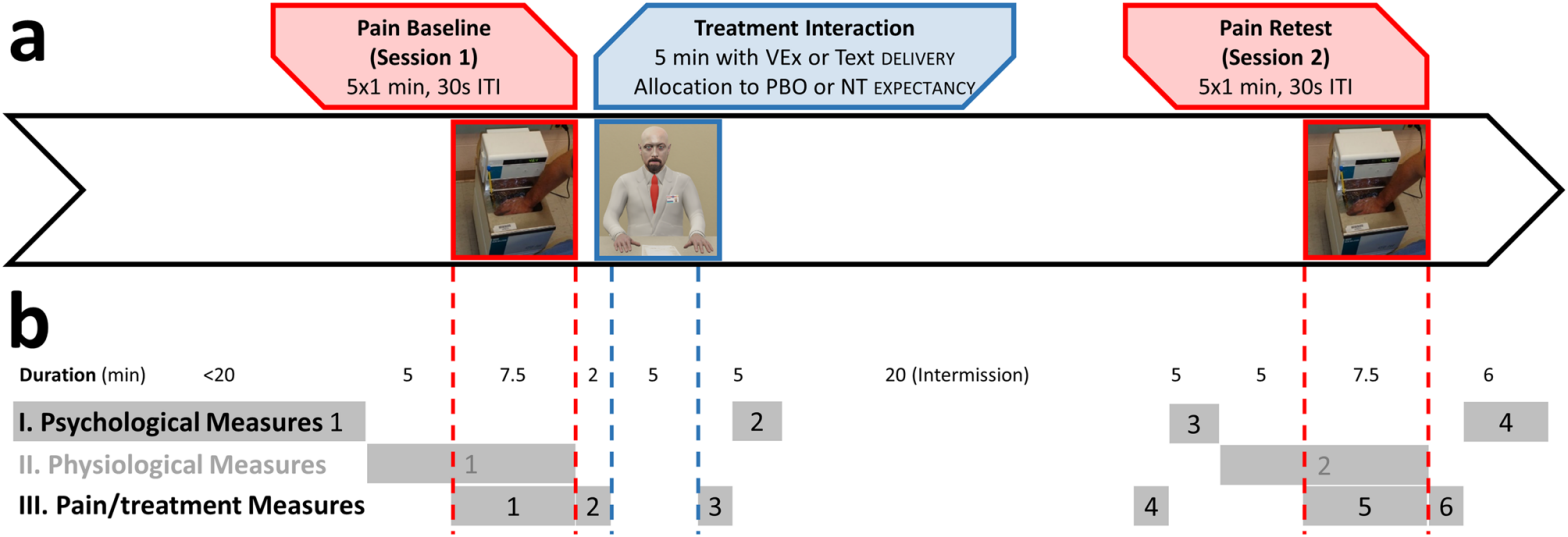
Experimental design and protocol. (**a**) Between pain baseline and retest including 5 hot water immersions, subjects were randomly assigned to Delivery and Expectancy conditions. Virtual experimenter screen captured in Unity. (**b**) Timing of measurements. Approximate durations are displayed in the upper row, grey areas indicate the duration of individual measurements, as described below. I. Four psychological measurements, 1 (General Information, IPC, LOT-R, PVAQ, FPQ-III, BFI-44, STAI-Trait, PHQ9, PHQ15, STAI-State 1, MDMQ-SF1), 2 (SPS, NMQ, EIC), 3 (STAI-State 2, MDMQ-SF2), 4 (Manipulation check, exploratory items). II. Physiological measures (data not shown). III. Pain/treatment measures, 1 (continuous ratings), 2 (retrospective ratings), 3 (side effects questionnaire, expectation of pain relief), 4 (side effects questionnaire), 5 (continuous ratings), 6 (retrospective ratings).

The experiment followed a 2 × 2 between-group design with nested 2 × 5 within-person repeated pain measurements (Fig. 1a). The repeated measurements were constituted by 5 baseline and 5 retest measurements (repeated measurement factors Immersion and Session; see “Experimental procedure” and “Pain protocol”). Between the two sessions, subjects were allocated to one of four groups defined by combination of the two between-group factors instruction delivery (factor Delivery) and expectancy manipulation (factor Expectancy). Delivery refers to the mode of communication employed during instruction delivery, either via Text or VEx (both conditions were accompanied by the same audio). Expectancy refers to whether a subject received a treatment introduced as an effective analgesic (a capsule really containing an inert substance, that is, a placebo/PBO), or no treatment (NT).

### Experimental procedure

The protocol was reviewed by Clemson University’s Institutional Review Board (vote IRB2016-349), and considered to conform to ethical standards. Subjects signed informed consent prior to participation, which excluded the fact that they might receive a deceptive instruction about their treatment (in PBO Expectancy condition). At conclusion of the study, all subjects were fully debriefed and had the opportunity to withdraw without repercussions. None of the subjects reported being upset after the deception was revealed, and none opted for withdrawal.

**Figure 2:**
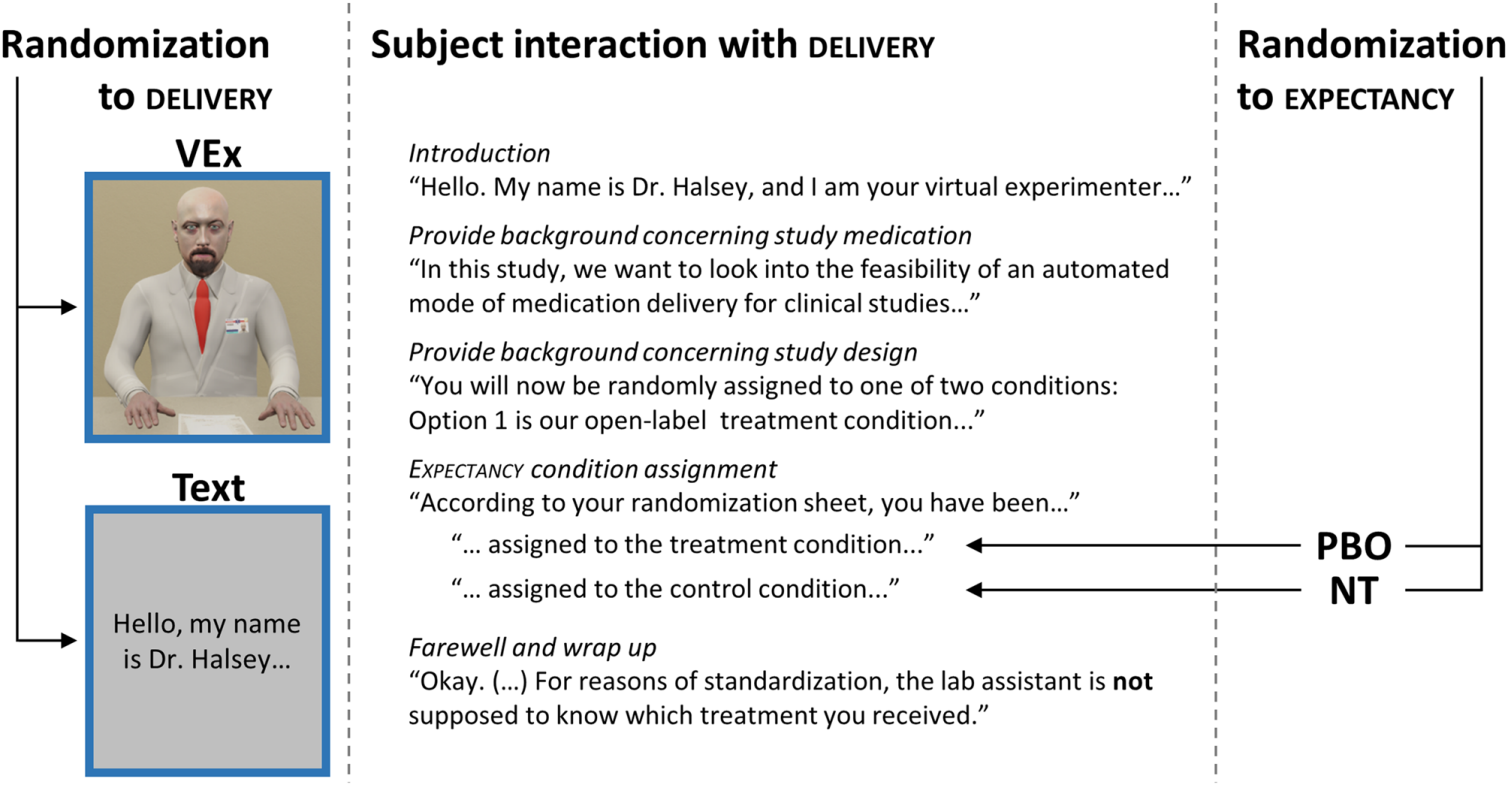
Treatment interaction with virtual experimenter or text/audio condition. Subjects randomly encountered either delivery condition. They then interacted with it (including confirmation of several sections, and option to ask FAQs). During the interaction, they were randomly assigned to either expectancy condition. Virtual experimenter screen captured in Unity.

Sequence of measurements and timings of the protocol are displayed in Fig. 1b. The experiment lasted about 1.5 h. It was conducted by a female experimenter (SB), in a room maintained at approximately 21 °C. Following instructions, preparations and baseline psychophysiological measurement, the baseline pain measurement started (session 1). Throughout the five one minute hot water immersions, subjects continually rated their pain on a touch screen. After session 1, subjects entered a neighboring room where they received the expectancy manipulation by allocation to one of four experimental groups on a large screen monitor (see Fig. 2 as well as “Experimental factor 1: instruction delivery” and “Experimental factor 2: expectancy manipulation”).

Importantly, in order to avoid corresponding biases, the human experimenter was unaware of group allocation in both experimental factors, and the subject was asked not to reveal allocation to the human experimenter. All subjects complied with this request, as per experimenter debriefing.

Subjects then returned to the main room for a 30 min intermission. Cover story for the intermission was for the supposed pain medication to take effect; the major intended side benefit was to allow for recovery of skin sensitivity. Subjects were instructed to avoid physical or stressful activities, but could otherwise spend the time ad libitum (e.g., by reading provided magazines). After the intermission, a retest pain measurement was conducted (session 2). Subjects were then debriefed and compensated for participation ($20, or $10 and course credit).

Various ratings and questionnaires were administered intermittently (Fig. 1b); heart rate was measured during the entire experiment. Note that psychophysiological measures were considered beyond the scope of this article to allow greater focus on behavioral measures.

### Pain protocol

We used a hot water bath (RTE-111, Neslab Instruments, Inc., Newington, NH) as an established method for painful stimulation^28,29^. The device is capable of circulating the water to avoid areas of cooler water insulating the skin. Subjects were asked to immerse their dominant hand in the water kept at a constant temperature of 47 °C, which is quite painful for most people^30^. Tonic heat pain has been successfully employed in the context of expectancy manipulations, with comparable temperatures^31,32^. In previous studies, we have established sufficient retest reliability to warrant the use of hot water in a baseline-corrected design^33^. Due to the relatively high intensity, it was emphasized that they could discontinue the experiment at any time.

Subjects were seated comfortably in front of a two monitor-setup (instruction screen and touch screen for ratings), and the water bath. They underwent a familiarization trial where they briefly immersed their right hand. The actual experimental sessions consisted of immersions of the hand for 1 min, removal for 30 s, and reinsertion, for a total of 5 immersions each session. Immersion and removal were prompted by verbal displays on screen, and concurrent audio signals.

Throughout the sessions, subjects continually rated their pain on a 0–100 digital visual analogue scale (VAS) using a touch screen. However, we avoided using second-by-second data in our analyses because this would lead to overly complex models. Instead, we averaged continuous pain ratings to obtain mean pain scores for each of the 2 × 5 immersions.

After each session, using the touch screen, they also gave a single retrospective rating on a numerical rating scale from 0 to 10 for pain intensity and pain unpleasantness to establish comparability to conventional non-continuous measures, and rated their desire for pain relief. Furthermore, they responded to a tolerance item intended to gauge the extent to which they believe they could have continued the stimulation (Supplementary Table S1).

### Experimental factor 1: instruction delivery

We created a Unity3D-based system for simulating the interactive text or virtual human (introduced as “Dr. Halsey”) based system (Unity Technologies, San Francisco, CA, USA). The simulation was displayed on a large screen 65 in. display, where the virtual human was rendered as a life size character to be perceived as sitting right across the table from the subject. Additional detail for the development of the VEx is provided in Supplementary Text S2. A Microsoft Kinect motion tracking system (Microsoft, Redmond, WA) was installed above the display and tracked the subjects’ head position in real-time. Using the head tracked position, Dr. Halsey’s head orientation was animated such that Dr. Halsey would maintain socially realistic face-to-face gaze behaviors.

A finite state machine was created so that the simulation would trigger the logical step-by-step instructions in the VEx or Text Delivery conditions. Both conditions were displayed with the same duration. The simulation in the Text Delivery was designed such that at each step of the instruction, the written instructions on the screen were accompanied by the respective audio recording. The corresponding words on the screen were highlighted as they were spoken, to increase the similarity in attentional focus and pacing between Text and VEx Delivery. In the VEx Delivery, Dr. Halsey executed discrete instruction sets of verbal and non-verbal behaviors based on the same state machine, with animation transitions between the end of a state and the beginning of the next. In this manner, Dr. Halsey delivered the instruction via naturalistic verbal and non-verbal behaviors.

At interaction points during instruction delivery, subjects were required to provide an input to a question such as “yes” or “no”, or select optional questions from an FAQ list, via a touchscreen interface. Either Delivery condition would provide appropriate responses to subject input before continuing with the step-by-step instructions. In the VEx condition when Dr. Halsey was awaiting input from the subjects, neutral life-like behaviors were displayed, such as breathing and small-scale head or hand movements.

### Experimental factor 2: expectancy manipulation

The Expectancy manipulation consisted of several steps intended to plausibly convey the notion that subjects have received an active treatment, when in fact, no active treatment was delivered. During instruction delivery, they were assigned to either the placebo (PBO) or the no-treatment control group (NT). Table 1 displays differences in Expectancy manipulation between the two groups, most notably the “medication” delivered to PBO but not to NT. Of note, all other aspects of the instruction (including e.g. duration, information about the drug) were identical between PBO and NT.

**Table 1:** Protocol differences between placebo and no-treatment Expectancy.

	Placebo condition (PBO)	No-treatment condition (NT)
Instruction (either by VEx or Text delivery)	“According to your randomization sheet, you have been assigned to the **treatment condition**. Please pick up the drug from the compartment and take it.[medication automatically dispensed] You will find a bottle of water there. Once you are finished, let me know.”	“According to your randomization sheet, you have been assigned to the **no treatment condition**. This means that you will not receive any medication, but will simply repeat the hot water test.”
Medication	Automatically dispensed, inert pill	None
Reinforcement 1	Questionnaire items addressing prior experience with the supposed medication, and its expected efficacy (expectation of pain relief), administered post treatment	n/a
Reinforcement 2	Questionnaire items supposedly assessing medication side effects, administered post treatment and post intermission	n/a

Beyond the core manipulation (inert medication versus no medication), instructions differed between groups, and the placebo group’s expectancies were reinforced on two occasions.

The supposed medication (actually an inert lactose placebo) was delivered by a custom-made pill dispenser controlled by an Arduino microprocessor (https://arduino.cc), which was triggered simultaneously with the respective part of instruction delivery.

### Psychological measures

Immediately after the intervention, subjects in the PBO expectancies responded to items concerning the treatment, which also served as a co-intervention (see Table 1, Reinforcement 1 and Reinforcement 2). Reinforcement 1 was framed as medication-related with two corresponding items (“Do you have experience with Acetaminophen?” and “If you have experience with it, how effective was Acetaminophen in alleviating your symptoms?”). These served as distractors for a third item assessing the expectation of pain relief (“Considering the pain stimulus you received, how effective do you expect it to be in reducing the pain?”, with possible responses “Not effective at all”, “Not very effective”, “Fairly effective” and “Very effective”).

Throughout the experiment, we used several questionnaires to assess psychological traits and states; references are provided in Supplementary Table S2. Among the trait questionnaires were the Internality, Powerful Others and Chance Scales (IPC), the Revised Life Orientation Test assessing optimism and pessimism (LOT-R), the Big Five Inventory-44 assessing extraversion, openness to experience, conscientiousness, neuroticism and agreeableness (BFI-44), the trait subscale of the State-Trait Anxiety Inventory (STAI), as well as the Pain Vigilance and Awareness Questionnaire (PVAQ) and the Fear of Pain Questionnaire III (FPQ-III). The state questionnaires included the state subscale of the STAI, and the Multidimensional Mood Questionnaire assessing positive versus negative affect, wakefulness versus tiredness, and calm versus nervousness (MDMQ).

Most of the questionnaires have been associated with placebo responding before^34–37^, but mood and BFI subscales were also intended for exploring affective influences on pain^38^.

Furthermore, we obtained ratings on form and content of the treatment interaction to compare VEx and Text Delivery. These measures included several scales established in virtual reality research. In particular, we assessed social presence with the Social Presence Scale (SPS), and co-presence, attentional allocation, perceived message understanding and perceived behavioral interdependence with the Networked Minds Questionnaire (NMQ). Furthermore, we used a questionnaire specifically constructed for this experiment, which assessed the VEx/Text deliveries’ believability, likeability and the extent to which it was perceived as compelling (Expectancy Induction Characteristics scale, EIC).

Psychological measures were subjected to a quality assessment^39,40^ (see Supplementary Table S3). After individual assessment of all flagged questionnaires responses, none of the subjects displayed sufficient grounds for global exclusion.

### Analysis

Due to the hierarchical nature of the data, we used a longitudinal hierarchical linear modeling (HLM) approach for analysis^41^. Among other advantages of HLM, imbalance in group sample sizes is unproblematic^42^. In the main HLM analyses, time variables (Immersion, Session) were entered as within-person variables. Second level (between-group) predictors (Delivery, Expectancy) were then entered into the base model to ascertain the effects of the experimental conditions. All HLM analyses were performed with random effects including intercept, Immersion (linear and squared), and Session. All repeatedly measured predictors were separated into their within-person component, and between-group components (aggregated to the person level).

Secondary analyses were then performed for two purposes: to assess possible moderation of the main results by various second level variables (e.g. personality traits), and to see if other first level variables (e.g. retrospective ratings) were changed following the intervention. For the first purpose, covariates were separately entered into the main analysis. They included subject doubt, desire for pain relief, expectation of pain relief, and several psychological traits. For the second purpose, the dependent variable (pain) used in the main analysis was replaced by the respective other first level variables. They included retrospective ratings (pain intensity, pain unpleasantness, and pain tolerance) as well as mood and anxiety questionnaires (STAI, MDMQ). For all secondary analyses, alpha correction was performed within the respective domains using effective number of tests (*M*_*eff*_) correction (Bonferroni correction adjusted for non-independence/correlation of predictors)^43^. The domains were pain measures, pain-related psychological traits, personality-related psychological traits, expectation of medication effects, Delivery-related measures, psychological states.

Analyses were performed with SPSS 22 (SPSS Inc., Chicago, IL, USA) and in MATLAB R2017b (The MathWorks Inc., Natick, MA, USA), exploratory mediation analyses using MATLAB’s VBA toolbox^44^. HLM analyses used Satterthwaite approximation for degrees of freedom. Significance level was set to 0.05. Descriptive statistics are reported as mean ± SE unless otherwise noted.

## Results

### Baseline comparability and additional sampling issues

**Figure 3:**
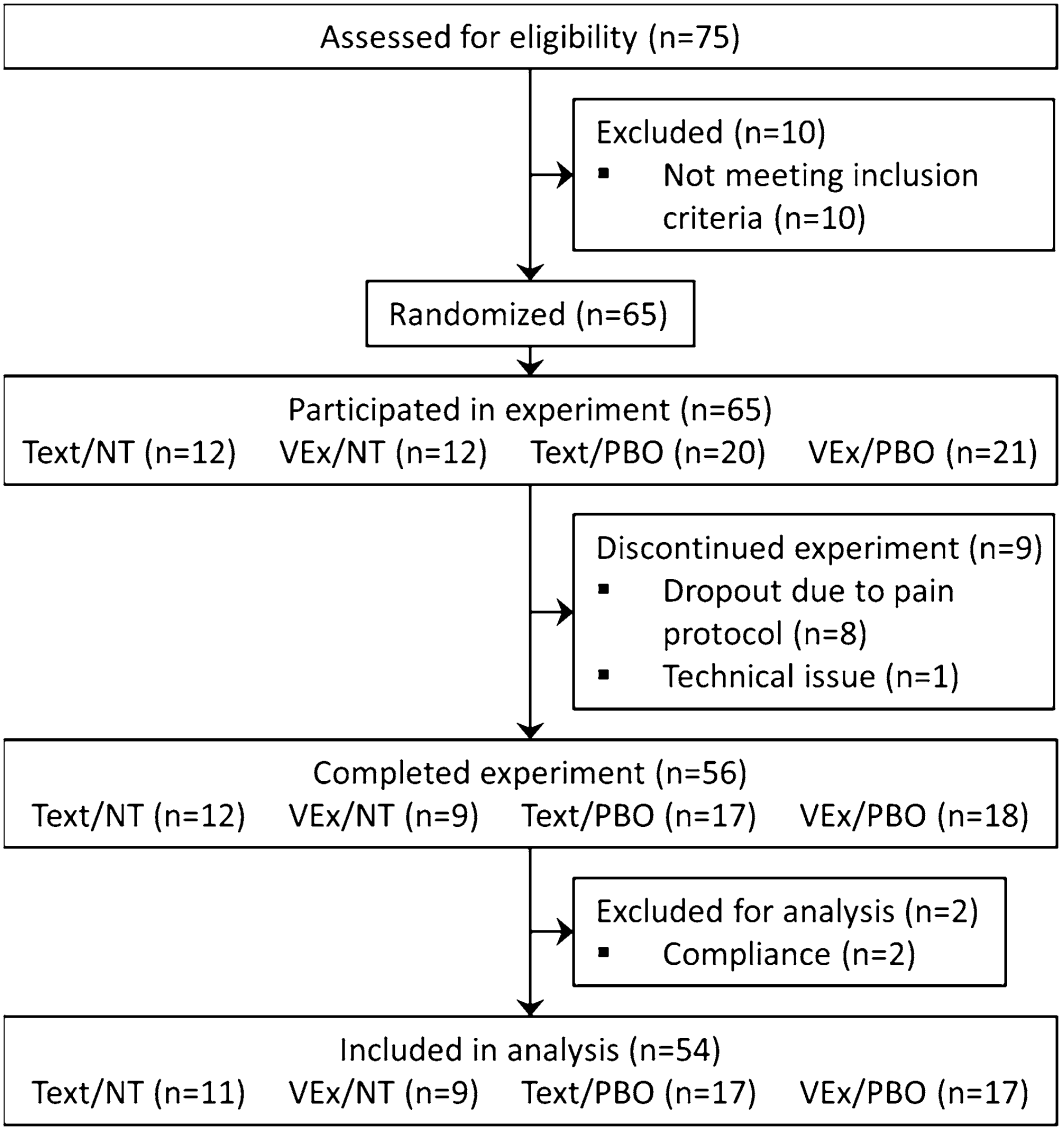
CONSORT statement for study enrollment. Attrition due to discontinued experiments was 14% (n = 9). Exclusions for compliance due to implausible core measures (pain ratings of zero) and antagonism (dozing off during baseline).

Fifty-four subjects were included in the study; the CONSORT flow diagram is provided in Fig. 3. Groups were comparable in all demographical and psychological state and trait measures assessed during baseline (see Supplementary Table S4). Completion of the psychological questionnaire battery took 16 ± 4 min (mean ± SD).

Allocation to the 2 × 2 experimental groups was asymmetric such that both placebo Expectancy conditions included more subjects than the control Expectancy conditions (n of Text/PBO 17, VEx/PBO 17, Text/NT 11, VEx/NT 9). This was done to perform subgroup analyses excluding subjects with doubts about the cover story. Unfortunately, we were not able to solve the issue of inferring a priori doubt about the instructions (that is, doubt existing before retest) from our a posteriori measurement (manipulation check after retest). Regardless, results do not differ whether including putatively unconvinced subjects or not (data not shown).

### Effects of immersion time on pain

**Figure 4:**
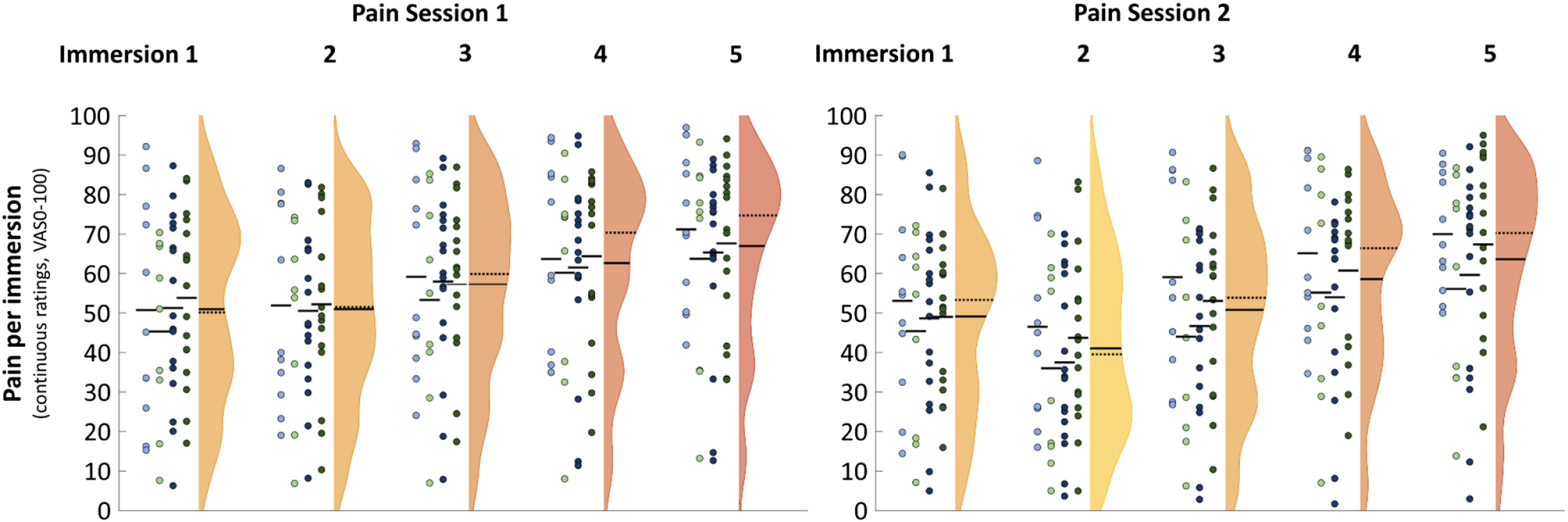
Pain ratings over both experimental sessions. Dot columns/colors indicate experimental groups (factor combinations of Delivery*Expectancy; column 1 light blue Text/NT, column 2 light green VEx/NT, column 3 blue Text/PBO, column 4 green VEx/PBO). Violins show full sample distribution, mean (solid line) and median (dotted line) per immersion.

The distribution of raw pain ratings is shown in Fig. 4. Interindividual differences were large, spanning almost the entire VAS. However, there are no conspicuous ceiling or floor effects.

The hierarchical linear model indicated that within-session pain ratings follow a quadratic time course: higher ratings in immersion 1, equal or lower ratings in immersion 2, and a roughly linear increase between immersions 2 and 5 (see Supplementary Table S5 for full results). Furthermore, pain VAS decreases between sessions (− 7.3 ± 1.5 VAS on average). Both findings are very robust and replicate our previous results^33^.

### Effect of experimental conditions

**Figure 5:**
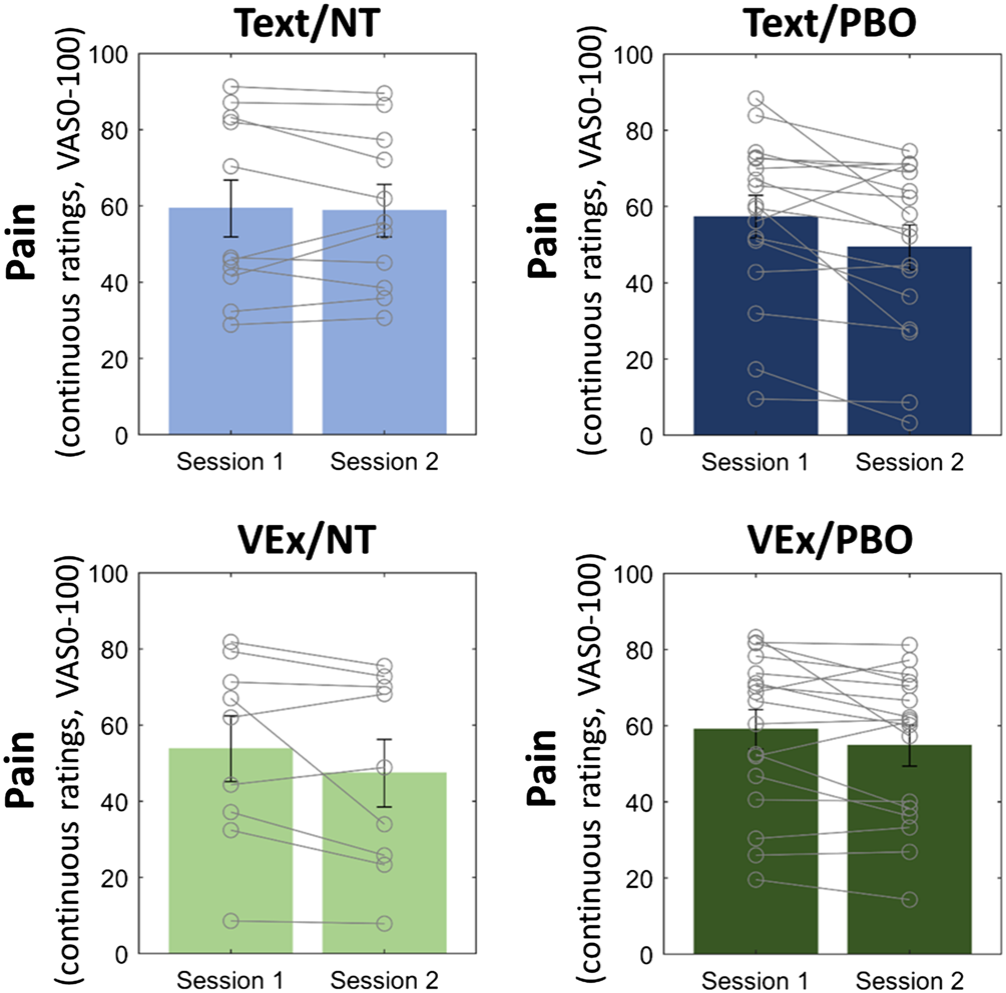
Change in mean pain ratings between session 1 and 2, by experimental condition (Delivery*Expectancy).

In addition to the base model (with first level-variables Immersion and Session), this model includes the effect of the two between-group experimental factors, Expectancy and Delivery (Table 2, Supplementary Table S6). Importantly, we see an interaction of Session*Expectancy*Delivery (p = 0.036). Inspecting pain changes between sessions (Fig. 5), we see that the interaction arises from the difference of the two Expectancy treatments in the Text Delivery (first column), that is, treatment differences arise according to our hypothesis (0.6 ± 3.1 VAS decrease in Text/NT, 8.0 ± 3.5 decrease VAS in Text/PBO; contrast t(507) = − 2.402, p = 0.017). Conversely, the VEx Delivery does not follow this intuitive mechanism, and there is no significant difference between the NT and PBO in this condition (6.4 ± 4.8 VAS decrease in VEx/NT, 4.2 ± 2.9 VAS decrease in VEx/PBO; contrast t(507) = 0.676, p = 0.5).

**Table 2:** Effects of immersion time and experimental conditions Delivery and Expectancy on continuous pain VAS ratings (hierarchical linear model).

	Estimate	SE	CI lower	CI upper	p
Session*Delivery	1.126	3.027	− 4.822	7.073	0.711
Session*Expectancy	− 3.857	3.135	− 10.016	2.302	0.222
Immersion*Session*Delivery	− 1.151	2.478	− 6.02	3.718	0.642
Immersion*Session*Expectancy	− 0.765	2.571	− 5.816	4.286	0.766
Session*Delivery*Expectancy	13.411	6.28	1.073	25.75	0.036

For brevity, only effects are displayed that a) included either one of the experimental conditions, and the session term, and b) are of lower or equal order than three-way interactions (higher order all n.s. p > 0.169). For the full model, see Supplementary Table S6.

*SE* standard error, *CI* 95% confidence interval.

## Secondary analyses

### Moderation of expectancy manipulation by desire for pain relief and expectation of pain relief

Average values for desire for pain relief were high (mean ± SD 6.13 ± 2.29 on a 0–10 scale; see Supplementary Table S4). Expectation of pain relief (0–3 scale) was assessed after the Expectancy manipulation, and only in the PBO Expectancies, with no mean difference between the Text and VEx Delivery (mean ± SD 1.41 ± 0.62 in Text, 1.59 ± 0.51 in VEx; two-sample t test t(32) = -0.910, p = 0.370).

Separately entering desire for pain relief (0–10 scale) and expectation of pain relief (0–3 scale) into the main analysis, a main effect of desire for pain relief indicated that a 1 point increase was associated with roughly 6.4 ± 1.1 higher VAS ratings over both sessions, across all conditions (p < 0.0001). Presumably, the causality here is that higher pain induces higher desire for its relief.

**Figure 6:**
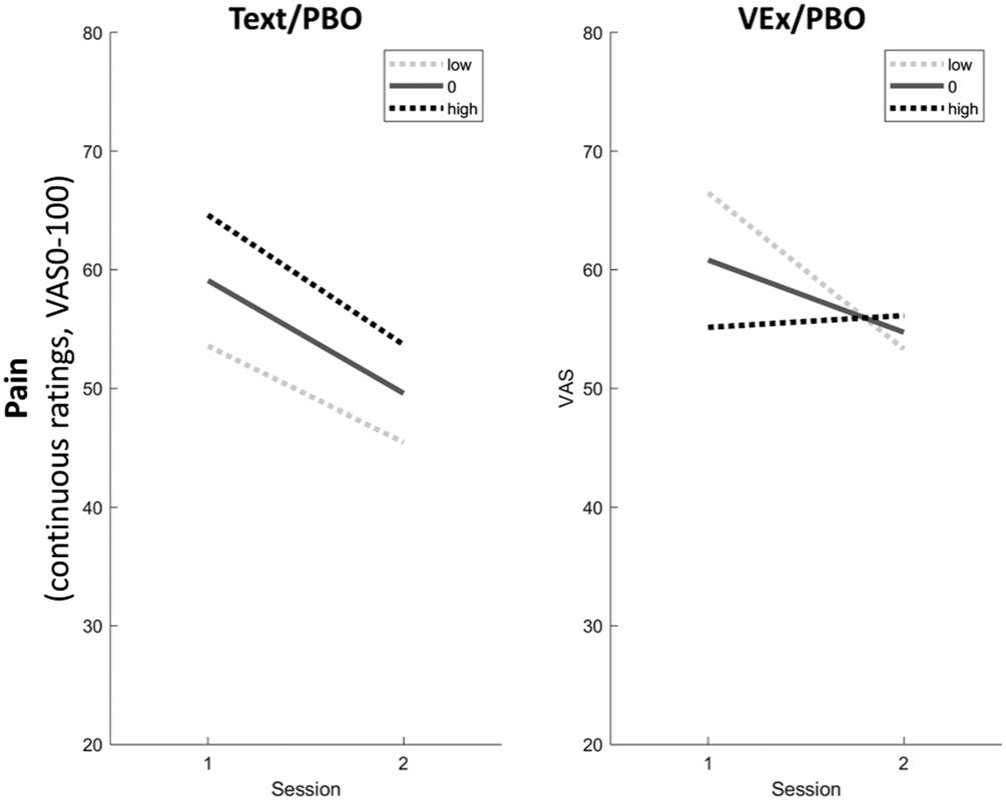
Moderation of the Delivery effect on pain by expectation of pain relief, controlling for desire for pain relief. In the Text Delivery (left panel), expectation has no effect (Session*Expectation p = 0.663, single contrast; the only conspicuous effects are due to baseline differences), whereas in the VEx Delivery (right panel), higher expectation was associated with lower pain improvement at retest (Session*Expectation p = 0.004, single contrast). Bright dotted line, low expectation of pain relief (− 1 standard deviation from group means); solid line, average expectation of pain relief; dark dotted line, high expectation of pain relief (+ 1 standard deviation from group means).

The expectation of pain relief significantly moderated pain change upon retest. Namely, we found a three-way interaction with session and Delivery, such that in the Text Delivery, expectation had no effect on pain relief, but in the VEx Delivery, higher expectation preceded lower pain relief (Fig. 6) (Session*Delivery*Expectancy p = 0.027).

Entering both desire for pain relief and expectation of pain relief simultaneously (for PBO only) did not change any of these results.

### Perception of the VEx compared to Text Delivery, and moderation by VEx perception

We found no significant correlations between either the hours spent on a computer or the hours spent using 3D technology, and any of the measures evaluating the VEx such as co-presence [highest r(24) = 0.28, p(uncorr.) = 0.172].

Individually entering each of the VEx measures as dependent variables in fixed effects models including Delivery and Expectancy as predictors, we found that Delivery had a significant main effect on co-presence after correction for *M*_*eff*_ = 7.094 [6.46 ± 1.64 higher NMQ in VEx Delivery; F(1,50) = 14.33, p(corr.) = 0.003]. This is unsurprising, as the co-presence scale contains items that strongly imply the presence of an embodied agent, and the visual perception of its attending the observer (e.g. “Dr. Halsey noticed me”, “I caught Dr. Halsey’s attention”). No other variable was significantly influenced by either Delivery or Expectancy.

Individually entering each of the VEx measures as moderators in the main hierarchical linear model did not yield any significant findings after correction for multiple comparisons (Supplementary Table S7).

### Moderation of expectancy manipulation by three pain-related subscales

Three pain-related subscales were entered into the main analysis individually, namely the PVAQ and the minor and severe pain subscales of the FPQ (Supplementary Table S8). We found no significant associations with or without correction for *M*_*eff*_ = 2.97 tests.

### Moderation of expectancy manipulation by ten personality-related subscales

Next, we individually entered the ten personality-related subscales from IPC, LOT-R, BFI and STAI into the main analysis. These analyses did not yield any significant findings after correction for multiple comparisons for *M*_*eff*_ = 9.162 [lowest p(corr.) = 0.054]. For exploratory reasons, we want to point out that trait optimism (LOT-R), the internality subscale of the IPC and the conscientiousness subscale of the BFI-44 show a moderation effect at uncorrected significance level (see Supplementary Table S9).

### Effect of experimental conditions on retrospective ratings

**Figure 7:**
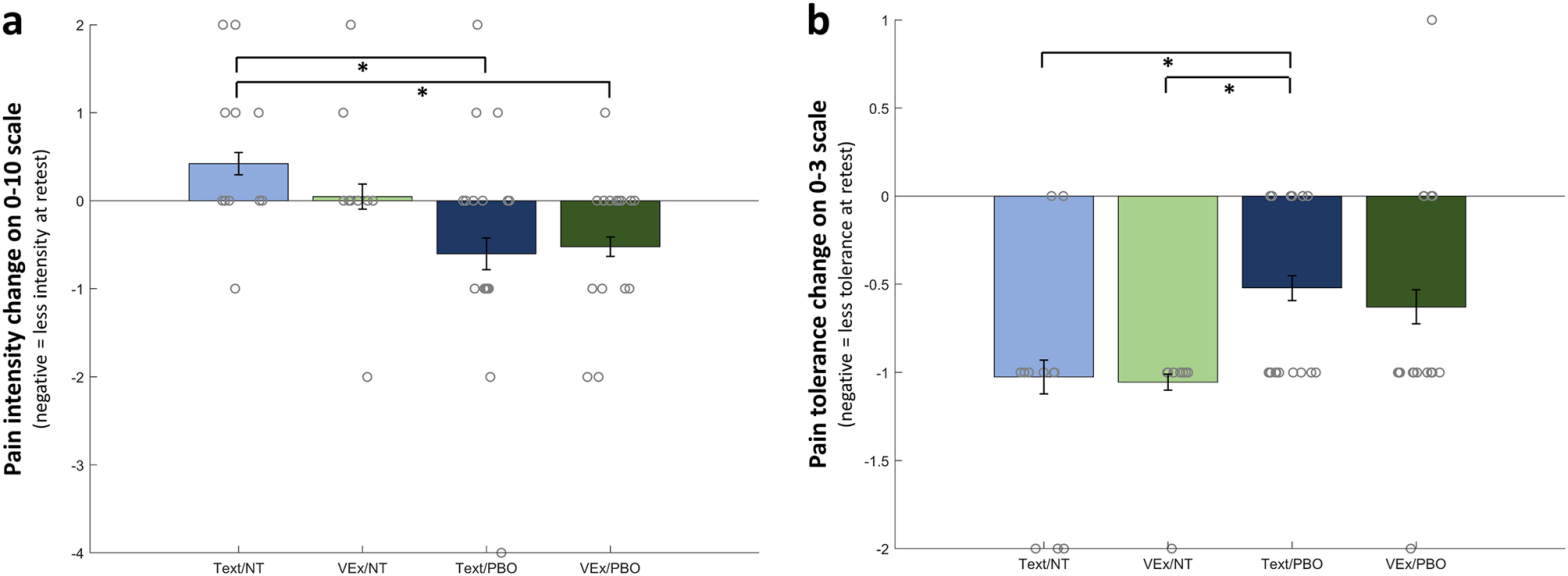
Changes in in retrospective ratings between baseline and retest for the four groups. Grey circles are single data points (score differences). Legend: *, p < 0.05. (**a**) Retrospective pain intensity ratings. (**b**) Retrospective pain tolerance ratings.

Next, we used the three retrospective ratings (0–10 numerical rating scales) individually as dependent variables, corrected for *M*_*eff*_ = 2.061 tests. For pain intensity, this yielded a significant effect of the experimental conditions (Session*Expectancy interaction, p(corr.) = 0.023). Single contrasts indicate that this effect was mostly driven by the difference between Text/NT (which actually reported increasing pain intensity between sessions) and the two PBO conditions (which reported decreasing pain intensity between sessions) (Fig. 7a). For the pain tolerance measure, a similar effect was found [Session*Expectancy interaction, p(corr.) = 0.016; Fig. 7b]. Note that model residuals did not show a deviation from normality (Shapiro–Wilk test p = 0.694), so we deemed the test as appropriate for the Guttman scale used for pain tolerance. We found no effect of experimental conditions for pain unpleasantness.

### Effect of experimental conditions on psychological state variables

**Figure 8:**
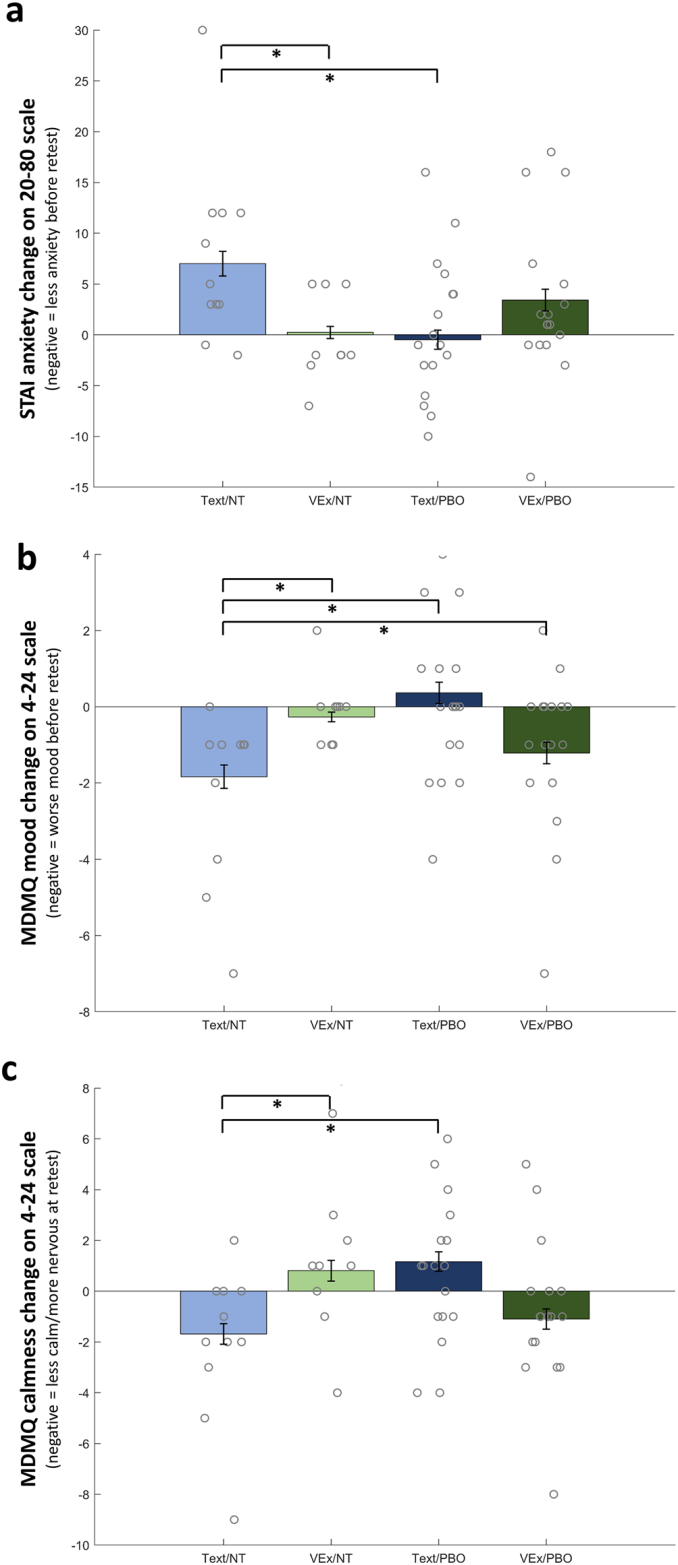
Changes in in psychological states between baseline and retest for the four groups, measured prior to the painful stimulation. Grey circles are single data points (score differences). (**a**) Change in STAI anxiety. (**b**) Change in MDMQ Good/Bad. (**c**) Change in MDMQ Calm/Nervous.

Four psychological state variables were entered as alternative dependent variables to assess the effect of the experimental conditions, corrected for *M*_*eff*_ = 2.598 tests. For the state anxiety scale of the STAI, contrary to the retrospective pain ratings where a clear main effect of PBO became visible (that is, significant Session*Expectancy interaction), an interaction of Session*Delivery*Expectancy reached significance [p(corr.) = 0.034; Fig. 8a]. This effect arose because in the Text/NT condition, a large increase in anxiety (~ 8 pts in 20–80 scale) occurred between the sessions which was absent in VEx/NT (single contrast p = 0.022) and Text/PBO (single contrast p = 0.022); however, no difference in anxiety changes were seen compared to the VEx/PBO condition (p = 0.149). Both the MDMQ Good/Bad scale and the MDMQ Calm/Nervous scale complement this finding, with three-way interactions on Good/Bad [p(corr.) = 0.017; Fig. 8b] and Calm/Nervous [p(corr.) = 0.015; Fig. 8c], driven by differences of Text/NT to VEx/NT and Text/PBO but not VEx/PBO.

As the effect of the experimental conditions on the three state variables closely mirrored those on continuous pain, we performed mediation analyses to see whether the pain changes were in any way related to the changes in anxiety. To do so, we entered difference scores of continuous pain between sessions as dependent variable, difference scores between the state variables as mediators, and performed separate analyses for each contrast of the experimental conditions. None of the analyses indicated significant mediation of pain change by any of the state variables (all p > 0.1 for Sobel tests of mediation, with p = 0.106 for mediation through the MDMQ Calm/Nervous subscale).

## Discussion

To investigate whether the delivery of treatment instructions could be fully standardized, while still preserving the necessary social interactions required for the generation of expectancy effects, we tested a virtual experimenter (VEx) against a text-only condition in a baseline-retest placebo manipulation. Overall, the automated delivery of a treatment was followed by substantial pain improvement. This is promising, because it means that some sources of bias in placebo research can be eliminated, such as bias from unblinding or lack of standardization.

Contrary to our hypotheses, we found no larger pain reduction when instructions were delivered by an embodied virtual agent, compared to a text-only condition. The main result was found in the Text Delivery with significant differences between PBO and NT group, such that pain decreased after placebo manipulation but not after no-treatment. However, this effect was absent in the VEx Delivery. Instead, placebo and no-treatment group both improved somewhat, but the effect was driven by a larger pain decrease in the no-treatment condition. This finding is intriguing, as it suggests that differences in the formal aspects of communication (embodied agent versus text), with identical explicit information content, could lead to effects as strong as “classical” expectancy effects observed under text-only, placebo versus no-treatment conditions.

Disappointingly, these results indicate that, in the current protocol, we have to reject our hypothesis of improved placebo responding after interacting with the VEx. This also implies that the intended “homogenization” of placebo effects through increased standardization failed to manifest. We note that the VEx was well-accepted but had no obvious superiority in terms of VR-related measures, as compared to the Text condition, beyond the co-presence measure attesting to the existence of an embodied agent. In other words, within the limited interaction employed in this experiment, measures such as attentional focus and message understanding did not benefit from the embodiment of the agent. Neither the measures concerning the VEx itself, nor its correlates in psychological state variables, offered straightforward explanations for these findings. It seems unlikely that the embodiment abolishes placebo responding altogether, given the effects on retrospective ratings discussed below, which directly compare to Text delivery. Granted the between-group design with comparatively large variances, it is likely that additional research is needed, possibly with larger sample sizes.

On state anxiety, nervousness and mood, the experimental conditions showed analogous effects to pain rating scales. It has been suggested that expectancy effects on pain partially work by affecting anxiety or stress^45–47^. In our case however, the absence of mediation effects suggests that either the effect on continuous pain and on psychological states were not observed in the same subjects, or that the association was too small for detection. Indeed, the study was not powered to conclusively perform this type of analysis. In either case, it is likely that complex psychological interactions are at the core of this finding, which is not exhaustively explained by expectancy.

For example, high expectation of pain relief, as measured in the PBO conditions only, had no effect in the Text Delivery, but led to a *lesser* pain decrease in the VEx Delivery. This is contrary to intuition and previously reported results^48,49^. Salient expectancy violations may have led to a contrast effect—instead of integrating the expectations of symptom relief (“assimilation”), a paradoxical symptom worsening occurs—or more generally, an aversive affective response^50^. Research documenting such effects exists on both the behavioral and neuronal level^51–53^. However, the identification of mechanisms is hindered by the fact that such effects do not reliably manifest. Therefore, the precise (experimental, much less clinical) conditions for when assimilation turns to a contrast effect are a matter of speculation^54^, for example, involving differences in sensory precision, attention, trust or prior experiences. Fittingly, if expectancy violations were to explain the effect of expectation of pain relief in the VEx group, they would fail to do so in the Text group where the PBO expectancy was assimilated.

Alternatively, while no corresponding data exists, we speculate that higher explicit expectations may have entailed a higher allocation of attentional resources to the pain experienced at retest. This would in turn make perception of the immediate sensory input more precise. As has been suggested^55^ and supported using a heat pain protocol^56^, when placebo hypoalgesia is conceptualized as a form of Bayesian integration, higher precision of sensory input would actually reduce placebo effects by reducing the impact of the expectancy prior.

Either interpretation does not explain the finding of comparatively large pain reduction in the VEx/NT condition. As we have argued before^34,57^, in control conditions, other factors such as a high internal locus of control may account for the extent to which aversive symptoms are experienced if only personal coping resources are available (i.e., no external attribution is possible, like on received medication). Our data somewhat corroborates this notion with a corresponding effect in the VEx/NT prior to alpha correction (higher internality leading to higher pain reduction), although internality had a parallel effect in the Text/PBO condition.

In any case, findings in the VEx condition are unlikely to be explained by an effect of distraction. While distraction can be a highly effective strategy to reduce pain^58^, the VEx manipulation was separated from the pain retest by a 30 min waiting period and not displayed concurrently to painful stimulation (as in^59^, for example). At best, a reminiscence of novelty may have persisted.

Furthermore, it is noteworthy that the described effects are only visible in continuous pain or indirect measures such as mood and anxiety. In the majority of studies of expectancy mechanisms, continuous pain measurements are not available, mostly due to more transient pain stimuli, and for methodological concerns that concurrent ratings may in itself be distracting. Instead, ratings are usually assessed after the respective pain trials. In our case, such retrospective ratings do not show the interaction between Expectancy and Delivery conditions, but flat effects of Expectancy on Session differences, across Delivery conditions. A number of studies have pointed out regularities in the way pain is remembered (for an overview, see ^60^). Most notably, this includes the peak-end rule^61,62^, which states that people tend to remember experiences mostly based on how they were perceived at their most intense, and at the end of the experience. In our study, the peak almost invariably coincides with the end. Therefore, one could argue that the continuous ratings including the entire previous pain trajectory merely introduce noise irrelevant for pain memory. Both pain experience and pain memory are important, one being a primary measure to reduce suffering, the other paramount for shaping future behavior, which is a crucial function of pain^63^. The latter function could involve, for example, dysfunctional behaviors implied by the fear-avoidance model^64^, or (non)adherence to treatments^62^ . An alternative interpretation of the disconnect between continuous and retrospective ratings may be that continuous ratings are less amenable to demand characteristics or similar response biases because of high working memory demands to provide them. Likewise, mood and anxiety are less transparently related to the expectancy manipulation and therefore may be less influenced by it. More dedicated studies are needed to address these issues.

We were satisfied to see that the pain protocol had sufficient variance between subjects that we could detect a significant effect of the Expectancy manipulation. We also opted against a within-person, cross-over design routinely employed in placebo research, which might have further reduced error variance, out of concerns for asymmetric carry-over effects^34^. The existence of a significant Expectancy effect is all the more noteworthy, as the simple expectancy manipulation we used (verbal suggestion of efficacy) is the most basic and arguably weakest form of manipulation employed in placebo mechanism studies^65,66^. While we have reinforced the placebo instruction intermittently (see Table 1), several other options exist to strengthen this protocol aspect now that basic efficacy was established^67^, the most obvious being to include some form of conditioning procedure (e.g.^49,66^). This is particularly important given that confidence intervals were comparatively large.

As an additional limitation, the study was not powered to investigate higher-level interactions of Expectancy and other determinants. For example, complex interactions between Expectancy, sex, optimism and anxiety may be involved in the generation of placebo effects^68^. The observed interactions with our experimental conditions support the notion that the identification of “simple” predictors of placebo responding^34^ may be a fool’s errand, with more interactional processes likely but harder to investigate^35,69^. Intriguingly, similarities and differences found between our experimental groups may eventually be explained by the way expectancies are formed in the first place, by different methods and in different people^5,70^.

In conclusion, we have demonstrated a fully standardized and automated procedure capable to deliver circumscribed treatments and hence generate expectancy effects. These responses did not scale with closer semblance to a human experimenter, as Delivery and Expectancy did not exhibit a synergistic effect as hypothesized. Instead, both had comparable effect size but seem to rely on different mechanisms. Putatively, a higher salience of the manipulation in the VEx Delivery was a driving factor for this divergence. Effects were moderated by expectation of pain relief and, on average, mimicked by those on psychological state variables.

Future directions should include replication in a more robust placebo protocol and/or a larger sample size. The addition of an actual human experimenter as a third mode of delivery, originally intended^18^ but scrapped for logistical reasons, should be strongly considered to investigate psychosocial determinants of treatment success. The interaction with the virtual agent could be expanded such that the VEx is also “present” during the pain sessions themselves. It would be very promising to eventually transfer this paradigm into an actual clinical trial to see how the effects of the treatment compare to its mode of delivery, with the ultimate aim of utilizing similar technology in clinical care itself^71^. Future manipulations could utilize internet-based and therefore highly scalable data collection such as Pavlovia (www.pavlovia.org) or Amazon Mechanical Turk (www.mturk.com). In its current state, systematic examination of overt traits such as age, sex, race or (non-)verbal behaviors in various healthy and patient populations could be an avenue to further the understanding of the mechanisms of placebo effects^72^, and how they affect ubiquitous symptoms like pain. Even in a purely experimental context, a strength of the VEx is that it allows for a tightly controlled manipulation of such overt characteristics of appearance and behavior^73^, and offers promise to the mitigation of bias in clinical research protocols.

## Supplementary information

Supplementary Information.
