# Supplementary material for: A virtual experimenter does not increase placebo hypoalgesia when delivering an interactive expectancy manipulation
Source: Sci Rep. 2020 Nov 23;10:20353. doi: 10.1038/s41598-020-77453-9 (PMC7684301; doi:10.1038/s41598-020-77453-9)
Supplement: Supplementary file 1 — Supplementary Information. [file 41598_2020_77453_MOESM1_ESM.docx]

A Virtual Experimenter does not increase placebo hypoalgesia
when delivering an interactive expectancy manipulation

Bjoern Horing, Sarah C. Beadle, Zachariah Inks, Andrew Robb, Eric R. Muth, Sabarish V. Babu

**Supplementary materials**

*Texts*

*Supplementary Text S1: Sample size estimation*

To determine optimal sample size for minimizing the chance of false positive and false negative findings, G*Power was employed^1^, using estimates for repeated measures ANOVA within/between-interactions as proxy. Granted our 2x2 conditions as well as 10 measurement repetitions per person, and assuming a small effect size of f=0.125 and a large correlation of 0.8 between measurements^2,3^, alpha threshold of p=.05 and power (1-beta) of 0.9, we have determined a total sample size of 40 (10 per group).

1. Faul, F., Erdfelder, E., Buchner, A. & Lang, A.-G. Statistical power analyses using G*Power 3.1: Tests for correlation and regression analyses. Behav. Res. Methods 41, 1149–1160 (2009)

2. Horing, B., Newsome, N. D., Enck, P., Babu, S. V. & Muth, E. R. A virtual experimenter to increase standardization for the investigation of placebo effects. BMC Med. Res. Methodol. 16, 84 (2016)

3. Horing, B., McCubbin, J. A., Moore, D. & Muth, E. R. Resting blood pressure differentially predicts time course in a tonic pain experiment. Psychophysiology 53, 1600–1607 (2016).

*Supplementary Text S2: Development of the Virtual Experimenter*

A 3D scan of one of the authors (BH) was first captured as the data source to be used in the modeling. The model was then refined using Blender (Blender Foundation, Amsterdam, Netherlands). Dr. Halsey was rigged with a complex skeletal animation system using MakeHuman (http://www.makehumancommunity.org). Facial expressions, gestures and lip-synched motions for the virtual human’s speech were captured using the FaceWare optical motion capture system (Austin, TX, USA). Used on the author’s speech behaviors, high fidelity shape key morph data was captured and applied to Dr. Halsey’s facial expressions, blinks, lip-synched speech and facial gestures (e.g. nodding). For each step of the instruction, these visual expressions (generated via a combination of motion captured shape key morph and skeletal animation data) were combined with the audio files of the speech input to generate appropriate speech behaviors. He was animated in Unity3D using the UMA framework. To preclude implicit biases, the verbal and non-verbal delivery of the instructions was carefully designed, choreographed and evaluated by an expert in interpersonal communications (JM) to ensure that the behaviors were of a neutral and standardized manner between Expectancy conditions.

*Tables*

*Supplementary Table S1*. Answering format for the tolerance item assessed immediately after the pain protocol. Answers are on a Guttman scale, with each description encompassing and expanding the previous answer. Since only full datasets were included, the scale effectively only includes item scores 0 to 3.

| Item score | Item description |
| --- | --- |
| 0 | I could have continued indefinitely |
| 1 | It was unpleasant, but I had no problem tolerating the pain |
| 2 | I thought about stopping the trial at some point, but did not seriously consider it |
| 3 | I seriously considered stopping the trial, but managed to endure |
| 4 | I stopped the trial, but I could have forced myself to continue |
| 5 | I stopped the trial because I felt compelled to do it/the pain became unbearable |

*Supplementary Table S2.* Reference list for psychological and virtual reality-related questionnaires.

| **Questionnaire** | **Reference** |
| --- | --- |
| Internality, Powerful Others and Chance Scales (IPC) | Levenson, H. Differentiating among internality, powerful others, and chance. in Research with the locus of control construct, Vol. 1 (ed. Lefcourt, H. M.) 15–63 (Academic Press, 1981). |
| Revised Life Orientation Test (LOT-R) | Scheier, M. F., Carver, C. S. & Bridges, M. W. Distinguishing optimism from neuroticism (and trait anxiety, self-mastery, and self-esteem): A reevaluation of the Life Orientation Test. J. Pers. Soc. Psychol. 67, 1063–1078 (1994). |
| Big Five Inventory-44 (BFI-44) | John, O. P., Donahue, E. M. & Kentle, R. L. The Big Five Inventory: Versions 4a and 54. (University of California, Institute of Personality and Social Research, 1991). |
| State-Trait Anxiety Inventory (STAI) | Spielberger, C. D., Gorsuch, R. L., Lushene, R. E., Vagg, P. R. & Jacobs, G. A. Manual for the State-Trait Anxiety Inventory. (Consulting Psychologists Press, 1970). |
| Pain Vigilance and Awareness Questionnaire (PVAQ) | McCracken, L. M. “Attention” to pain in persons with chronic pain: A behavioral approach. Behav. Ther. 28, 271–284 (1997). |
| Fear of Pain Questionnaire III (FPQ-III) | McNeil, D. W. & Rainwater, A. J. Development of the Fear of Pain Questionnaire-III. J. Behav. Med. 21, 389–410 (1998). |
| Multidimensional Mood Questionnaire (MDMQ) | Steyer, R., Schwenkmezger, P., Notz, P. & Eid, M. Der Mehrdimensionale Befindlichkeitsfragebogen (MDBF). Handanweisung. (Hogrefe, 1997). |
| Social Presence Scale (SPS) | Bailenson, J. N., Blascovich, J., Beall, A. C. & Loomis, J. M. Equilibrium revisited: Mutual gaze and personal space in virtual environments. Presence Teleoperators Virtual Environ. 10, 583–598 (2001). |
| Networked Minds Questionnaire (NMQ) | Harms, C. & Biocca, F. Internal consistency and reliability of the Networked Minds Social Presence Measure. in Proceedings of Presence Conference 246–251 (2004). |
| Expectancy Induction Characteristics scale (EIC) | Horing, B., Newsome, N. D., Enck, P., Babu, S. V. & Muth, E. R. A virtual experimenter to increase standardization for the investigation of placebo effects. BMC Med. Res. Methodol. 16, 84 (2016). |

*Supplementary Table S3.* Number of subjects flagged for suspicious response behavior, and number of excluded subjects after individual assessment. Subjects could be flagged for more than one reason. In total, 18 subjects were flagged for at least one of the reasons; none displayed sufficient grounds for exclusion. The column “parameter description” includes a short characterization of the metric behind the respective flags. Flags were raised when comparing individual parameters to the rest of the sample – for example, if a person’s perseveration in a questionnaire was above 3.1SD different than that of others in the sample.

| Flagged for | Parameter description | N flagged | N excluded |
| --- | --- | --- | --- |
| Perseverance outliers | High number of same answers in a row, within a questionnaire | 8 | 0 |
| Frequent changes outliers | High number of answer changes within a questionnaire | 2 | 0 |
| Psychometric synonym outliers | Low or negative correlation between items with high positive correlation in the sample | 1 | 0 |
| Psychometric antonym outliers | Low or positive correlation between items with high negative correlation in the sample | 0 | n/a |
| Multivariate outliers | Large average distance to the sample in questionnaire scores | 12 | 0 |
| Univariate outliers | Large outlier in individual questionnaire score | 3 | 0 |

*Supplementary Table S4*. Descriptive statistics for demographical, psychological state and trait, and psychophysiological measures. No differences were found, with the exception of the internality subscale of the IPC (with lower values in the Text/NT condition), the openness subscale of the BFI (with higher values in VEx/NT and Text/PBO). Since safeguards in the protocol rule out randomization failure, we can offer no explanation except random chance for this finding. p values reported for Delivery (D), Expectancy (E), and Delivery*Expectancy (DE) terms.

|  |  | **Group** |  |  |  | **p(D)** | **p(E)** | **p(D*E)** |
| --- | --- | --- | --- | --- | --- | --- | --- | --- |
|  |  | **Text** | **VEx** | **Text** | **VEx** |  |  |  |
|  | **Mean±SD** | **NT** | **NT** | **PBO** | **PBO** |  |  |  |
| **Basic characteristics** |  |  |  |  |  |  |  |  |
| N | 54 | 11 | 9 | 17 | 17 |  |  |  |
| N sex (w\|m) | 29\|25 | 6\|5 | 5\|4 | 7\|10 | 11\|6 |  |  |  |
| Age | 19.6±2.6 | 19.6 | 19.7 | 19.9 | 19.4 | 0.773 | 0.995 | 0.743 |
| Interact w computers (h/day) | 4.4±2.2 | 4.5 | 4.3 | 3.9 | 5 | 0.461 | 0.950 | 0.317 |
| Exposed to 3D tech (h/day) | 1.8±3.1 | 1.5 | 0.5 | 2.9 | 1.8 | 0.239 | 0.128 | 0.915 |
| **Psychological traits** |  |  |  |  |  |  |  |  |
| IPC Internality | 36.8±4.4 | 33.1 | 39.2 | 37.4 | 37.3 | **0.011** | 0.315 | **0.010** |
| IPC PowerfulOthers | 47.3±4.3 | 49.1 | 48 | 46.4 | 46.6 | 0.744 | 0.098 | 0.571 |
| IPC Chance | 48.2±4.0 | 49.2 | 48.4 | 48.2 | 47.5 | 0.533 | 0.393 | 0.989 |
| LOTR total | 8.2±4.4 | 6.3 | 8.2 | 8.9 | 8.8 | 0.451 | 0.203 | 0.423 |
| PVAQ total | 37.9±10.5 | 37.5 | 38.1 | 38.4 | 37.6 | 0.974 | 0.948 | 0.828 |
| FPQ Minor pain | 15.3±3.9 | 15.5 | 14 | 16.2 | 14.8 | 0.188 | 0.498 | 0.952 |
| FPQ Severe pain | 21.5±4.7 | 22.1 | 23.2 | 21.9 | 19.7 | 0.677 | 0.170 | 0.207 |
| BFI44 Extraversion | 26.6±6.7 | 25.3 | 28.9 | 27.1 | 25.8 | 0.550 | 0.730 | 0.209 |
| BFI44 Agreeableness | 35.2±4.0 | 34.9 | 35.8 | 36.3 | 34.1 | 0.551 | 0.884 | 0.179 |
| BFI44 Conscientiousness | 34.7±5.3 | 34.1 | 35.1 | 34.8 | 34.8 | 0.742 | 0.916 | 0.742 |
| BFI44 Neuroticism | 22.2±6.8 | 21.5 | 20 | 21.8 | 24.4 | 0.787 | 0.240 | 0.287 |
| BFI44 Openness | 35.6±6.0 | 33.4 | 39 | 37.1 | 33.7 | 0.483 | 0.622 | **0.008** |
| STAI-T total | 42.0±6.8 | 41.7 | 40.6 | 42.5 | 42.5 | 0.755 | 0.492 | 0.778 |
| **Psychological states baseline** |  |  |  |  |  |  |  |  |
| STAI-S total | 32.3±9.5 | 30.8 | 30.4 | 34.1 | 32.4 | 0.705 | 0.339 | 0.808 |
| MDMQ Good/Bad | 19.3±2.9 | 19.9 | 19.9 | 18.5 | 19.4 | 0.632 | 0.257 | 0.615 |
| MDMQ Awake/Tired | 15.2±3.5 | 16.3 | 16.3 | 14.8 | 14.2 | 0.811 | 0.070 | 0.763 |
| MDMQ Calm/Nervous | 18.3±2.9 | 19.2 | 17.7 | 17.9 | 18.5 | 0.603 | 0.793 | 0.198 |
| **Baseline pain ratings** |  |  |  |  |  |  |  |  |
| Pain VAS (mean of 5 immersions) | 57.7±21.7 | 59.3 | 53.8 | 57.3 | 59 | 0.761 | 0.796 | 0.566 |
| NRS Intensity | 6.4±2.0 | 6.3 | 6.7 | 6.2 | 6.4 | 0.583 | 0.759 | 0.890 |
| NRS Unpleasantness | 6.8±1.9 | 7.3 | 7.3 | 6.4 | 6.6 | 0.788 | 0.165 | 0.874 |
| NRS Desire for pain relief | 6.1±2.3 | 6 | 6.6 | 6.2 | 5.9 | 0.879 | 0.743 | 0.496 |
| NRS Tolerance | 1.9±0.8 | 2.2 | 1.4 | 1.9 | 1.9 | 0.084 | 0.742 | 0.084 |

*Supplementary Table S5.* Effects of immersion time on continuous pain VAS ratings. This is the base hierarchical linear model that was included in most later analyses, e.g. those investigating moderation of other predictor variables.

| **Term** | **Estimate** | **SE** | **CI lower** | **CI upper** | **p** |
| --- | --- | --- | --- | --- | --- |
| Intercept | 52.828 | 2.874 | 47.181 | 58.474 | <0.0001 |
| Immersion | 8.611 | 0.729 | 7.179 | 10.043 | <0.0001 |
| Session | -7.282 | 1.521 | -10.27 | -4.295 | <0.0001 |
| Immersion^2 | 1.164 | 0.172 | 0.825 | 1.503 | <0.0001 |
| Immersion*Session | 3.725 | 1.229 | 1.311 | 6.139 | 0.003 |
| Immersion*Immersion^2 | -1.202 | 0.17 | -1.537 | -0.868 | <0.0001 |
| Session*Immersion^2 | 1.133 | 0.345 | 0.455 | 1.81 | 0.001 |
| Immersion*Session*Immersion^2 | -1.026 | 0.341 | -1.695 | -0.357 | 0.003 |

*Supplementary Table S6.* Effects of immersion time and experimental conditions on continuous pain VAS ratings. Rows in bold are included in table in manuscript proper.

| **Term** | **Estimate** | **SE** | **CI lower** | **CI upper** | **p** |
| --- | --- | --- | --- | --- | --- |
| Intercept | 52.676 | 2.932 | 46.915 | 58.438 | <0.0001 |
| Immersion | 8.634 | 0.737 | 7.185 | 10.082 | <0.0001 |
| Session | -7.43 | 1.513 | -10.402 | -4.458 | <0.0001 |
| Immersion^2 | 1.167 | 0.174 | 0.826 | 1.508 | <0.0001 |
| Delivery | -0.876 | 5.869 | -12.407 | 10.655 | 0.882 |
| Expectancy | -0.409 | 6.075 | -12.344 | 11.526 | 0.947 |
| Immersion*Session | 3.705 | 1.238 | 1.273 | 6.137 | 0.003 |
| Immersion*Immersion^2 | -1.213 | 0.172 | -1.55 | -0.876 | <0.0001 |
| Session*Immersion^2 | 1.154 | 0.347 | 0.472 | 1.836 | 0.001 |
| Immersion*Delivery | 0.75 | 1.476 | -2.15 | 3.649 | 0.612 |
| **Session*Delivery** | **1.126** | **3.027** | **-4.822** | **7.073** | **0.711** |
| Immersion^2*Delivery | -0.014 | 0.347 | -0.696 | 0.669 | 0.969 |
| Immersion*Expectancy | -1.066 | 1.53 | -4.072 | 1.94 | 0.486 |
| **Session*Expectancy** | **-3.857** | **3.135** | **-10.016** | **2.302** | **0.222** |
| Immersion^2*Expectancy | 0.27 | 0.36 | -0.437 | 0.977 | 0.453 |
| Delivery*Expectancy | 12.599 | 12.17 | -11.311 | 36.509 | 0.305 |
| Immersion*Session* Immersion^2 | -1.03 | 0.343 | -1.704 | -0.356 | 0.003 |
| **Immersion*Session* Delivery** | **-1.151** | **2.478** | **-6.02** | **3.718** | **0.642** |
| Immersion*Immersion^2* Delivery | -0.143 | 0.343 | -0.818 | 0.532 | 0.678 |
| Session*Immersion^2* Delivery | -0.422 | 0.695 | -1.787 | 0.944 | 0.544 |
| **Immersion*Session* Expectancy** | **-0.765** | **2.571** | **-5.816** | **4.286** | **0.766** |
| Immersion*Immersion^2* Expectancy | 0.112 | 0.356 | -0.587 | 0.812 | 0.753 |
| Session*Immersion^2* Expectancy | 0.477 | 0.719 | -0.936 | 1.89 | 0.508 |
| Immersion*Delivery* Expectancy | -1.232 | 3.066 | -7.255 | 4.791 | 0.688 |
| **Session*Delivery* Expectancy** | **13.411** | **6.28** | **1.073** | **25.75** | **0.036** |
| Immersion^2*Delivery* Expectancy | -0.217 | 0.721 | -1.633 | 1.198 | 0.763 |
| Immersion*Session* Immersion^2*Delivery | 0.489 | 0.687 | -0.861 | 1.838 | 0.477 |
| Immersion*Session* Immersion^2*Expectancy | 0.576 | 0.712 | -0.823 | 1.976 | 0.419 |
| Immersion*Session* Delivery*Expectancy | 0.257 | 5.153 | -9.866 | 10.381 | 0.96 |
| Immersion*Immersion^2* Delivery*Expectancy | 0.784 | 0.714 | -0.618 | 2.187 | 0.272 |
| Session*Immersion^2* Delivery*Expectancy | -1.984 | 1.441 | -4.816 | 0.847 | 0.169 |
| Immersion*Session* Immersion^2*Delivery* Expectancy | 0.672 | 1.427 | -2.132 | 3.477 | 0.638 |

Supplementary Table S7. No moderation of Delivery and Expectancy results by VEx evaluation variables. The displayed p values are uncorrected; the corrected alpha level is p=0.0070481 (M_eff_=7.094), at which none of the terms prevail. Legend: ^†^, significant at uncorrected alpha (p<0.05).

| Predictor | Session*Delivery* Predictor | Session*Expectancy* Predictor | Session*Delivery* Expectancy*Predictor |
| --- | --- | --- | --- |
| VEx Message | 0.531 | 0.139 | 0.009^†^ |
| VEx Likeability | 0.168 | 0.052 | 0.011^†^ |
| VEx Compelling | 0.66 | 0.074 | 0.615 |
| SPSR total | 0.887 | 0.810 | 0.884 |
| NMQ Co-presence | 0.177 | 0.440 | 0.213 |
| NMQ Attention allocation | 0.674 | 0.371 | 0.408 |
| NMQ Perc. msg. understanding | 0.74 | 0.901 | 0.627 |
| NMQ Perc. behav. interdep. | 0.952 | 0.738 | 0.343 |

*Supplementary Table S8*. No moderation of Session, Delivery and Expectancy effects by pain vigilance and awareness, or fear of pain. The displayed p values are *uncorrected*; the corrected alpha level is p=0.016832 (*M_eff_*=2.97).

| Predictor | Session*Predictor | Session*Delivery* Predictor | Session*Expectancy* Predictor | Session*Delivery* Expectancy*Predictor |
| --- | --- | --- | --- | --- |
| PVAQ total | 0.193 | 0.67 | 0.178 | 0.297 |
| FPQ Minor | 0.621 | 0.306 | 0.47 | 0.666 |
| FPQ Severe pain | 0.055 | 0.214 | 0.325 | 0.285 |

*Supplementary Table S9*. No moderation of Delivery and Expectancy results by pain vigilance and awareness, or fear of pain. The displayed p values are *uncorrected*; the corrected alpha level is p=0.0054571 (*M_eff_*=9.162). Legend: ^†^, significant at uncorrected alpha (p<0.05).

| Predictor | Session*Predictor | Session*Delivery* Predictor | Session*Expectancy* Predictor | Session*Delivery* Expectancy*Predictor |
| --- | --- | --- | --- | --- |
| IPC Internality | 0.402 | 0.491 | 0.54 | 0.011^†^ |
| IPC PowerfulOthers | 0.051 | 0.187 | 0.447 | 0.896 |
| IPC Chance | 0.084 | 0.134 | 0.839 | 0.808 |
| LOTR total | 0.833 | 0.006**^†^** | 0.855 | 0.287 |
| BFI44 Extraversion | 0.816 | 0.232 | 0.963 | 0.431 |
| BFI44 Agreeableness | 0.791 | 0.697 | 0.725 | 0.28 |
| BFI44 Conscientiousness | 0.199 | 0.006**^†^** | 0.079 | 0.97 |
| BFI44 Neuroticism | 0.396 | 0.292 | 0.605 | 0.498 |
| BFI44 Openness | 0.832 | 0.162 | 0.905 | 0.293 |
| STAI-T total | 0.172 | 0.245 | 0.602 | 0.187 |
